# Defending a mean arterial pressure in the intensive care unit: Are we there yet?

**DOI:** 10.1186/s13613-018-0463-x

**Published:** 2018-12-03

**Authors:** Ashish K. Khanna

**Affiliations:** Center for Critical Care, Department of General Anesthesiology and Outcomes Research, Anesthesiology Institute, Cleveland Clinic, 9500 Euclid Avenue, G-58, Cleveland, OH 44195 USA

I read with great interest, the work of Jean-Louis Vincent and colleagues ‘Mean Arterial Pressure and Mortality in Patients with Distributive Shock: A Retrospective Analysis of the MIMIC-III Database’ [1]. I congratulate the authors on this work that highlights our current standards of care when it comes to protecting our critically ill patients against hypotension, as defined by a threshold mean arterial pressure (MAP). The surviving sepsis campaign guidelines recommend that vasopressors be titrated to a MAP of at least 65 mmHg while resuscitating septic shock [2]. Does this mean that a MAP of 65 mmHg ‘protects’ the critically ill patient from organ system injury? Or is this MAP of 65 mmHg the ‘one size fits all’ for all our patients? The only landmark-randomized control trial in this space has been performed by Asfar and colleagues. They randomized patients with septic shock to a low MAP target (65–70 mmHg) or a high MAP target (80–85 mmHg). The authors reported that average MAP values in the low MAP target arm were above 70 mmHg and in the high MAP target arm were closer to 85 mmHg. However, the allocation of patients to either MAP group did not influence 28-day or 90-day mortality [3]. Several new questions have been asked recently, and within the limitations of retrospective data, these thresholds may be different and higher than a MAP of 65 mmHg as the only perceived ‘magic number’ [4].

There are several merits of the authors work. Firstly, the database in question—the Medical Information Mart for Intensive Care (MIMIC-III) is a well validated, robust dataset with about 50,000 ICU admissions and related variables. Secondly, the authors work is novel—while we all believe that we know that hypotension in the ICU is bad and we think that we are doing a good job defending a MAP of 65 mmHg, the results reported here make it clear that we are not. As reported by Jean-Louis Vincent’s group, more than 60% of this population in question had a continuous MAP of less than 65 mmHg of at least 2 h to up to 4 h of duration. (This was after being exposed to a minimum of 6 h of preceding vasopressor therapy to stabilize blood pressure). The story is not very different when lower thresholds of defending MAPS are analyzed as well. In addition, the authors elegantly show a progressive increase in ICU mortality as the duration of exposure to MAPS less than 65, 60 and 55 mmHg is lengthened.

Where does this work take us next? What MAP should I defend in the ICU, and how should I best do it? This analysis of MIMIC-III does have its own limitations. The biggest one is the lack of granularity of blood pressure data that were recorded about once per hour. This is something that most hemodynamic analytics in the ICU are faced with currently, as we report only nurse-verified ICU blood pressures which may be few and far between, and indeed may be more for only the perceived sicker patients. We as a critical care research community need to develop an effective system of conserving continuously generated hemodynamic data (or monitor data) with an inherent mechanism to filter out the noise and artifacts. This is the only way that we will be able to effectively report associations with outcomes with some degree of certainty. The other opportunities in this work concern vasopressors, and maybe something that the authors would want to reconsider for the future. It would be fascinating to analyze a MAP of 65 mmHg and even higher and lower thresholds, in the context of increasing vasopressors, specifically the use of catecholamines only versus a more multi-modal balanced approach using vasopressin and other newer analogs as well. Furthermore, the utilization of high-dose vasopressors has been associated with 50–80% 30-day and 90-day mortality, and balance of adequacy of blood pressure versus the necessity of loading vasopressors is an evolving argument [5]. Finally, the authors did adjust for several confounders (age, sex, sepsis, highest catecholamine dose, baseline mechanical ventilation status, renal replacement, SOFA score, baseline creatinine, lactate and albumin levels)—however, even the best-adjusted dataset may never be free from hidden confounding, and the skeptic will always be concerned that the higher mortality reported with more duration of hypotension less than a MAP of 65 mmHg is actually a reflection of the underlying disease state more than the direct damage done by hypotension.

The need of the hour is larger, more granular hemodynamic datasets such as these that can allow us to precisely see what we are doing with our defense of blood pressures in the ICU. Outcomes such as mortality are important and serious; however, other critical outcomes such as myocardial injury after non-cardiac surgery also need to be considered. Clearly, if the perceived ‘magic number’ is a mean arterial pressure of 65 mmHg, we are not quite there yet, and we are not doing a good job in terms of defending it. If the number is higher than 65 mmHg, then the situation may be even more worrisome. There remain several unanswered questions in religiously defining and appropriately defending a blood pressure in the ICU.
